# Ocular motility changes after inferomedial wall and balanced medial plus lateral wall orbital decompression in Graves’ orbitopathy: a randomized prospective comparative study

**DOI:** 10.6061/clinics/2021/e2592

**Published:** 2021-03-30

**Authors:** Cristiane de Almeida Leite, Thaís de Sousa Pereira, Jeane Chiang, Rodrigo Bernal Moritz, Allan Christian Pieroni Gonçalves, Mário Luiz Ribeiro Monteiro

**Affiliations:** Laboratorio de Investigacao em Oftalmologia (LIM 33), Divisao de Oftalmologia, Faculdade de Medicina FMUSP, Universidade de Sao Paulo, Sao Paulo, SP, BR

**Keywords:** Graves Ophthalmopathy, Exophthalmos, Decompression Surgical, Strabismus, Diplopia

## Abstract

**OBJECTIVES:**

To compare the surgical outcomes of inferomedial wall orbital decompression (IM-OD) and balanced medial plus lateral wall orbital decompression (ML-OD) in patients with inactive Graves’ orbitopathy (GO) with regard to exophthalmos reduction and ocular motility abnormalities.

**METHODS:**

Forty-two patients with inactive GO eligible for OD were randomly assigned to either the IM-OD or ML-OD groups. Pre and postoperative evaluations included Hertel exophthalmometry, sensory, and motor extraocular motility assessment, standardized photographs in the nine gaze positions, and computed tomography (CT) of the orbits. ClinicalTrials.gov: NCT03278964.

**RESULTS:**

Exophthalmometry reduction was statistically significant in both groups (*p*<0.001), but was greater in the ML-OD group (*p*=0.010). New-onset esotropia occurred in 11.1% and 23.5% of patients who underwent IM-OD and ML-OD, respectively, with no statistically significant difference in the frequency of pre and postoperative strabismus in either group. The mean increase in preoperative esotropia was 24±6.9 and 12±8.8 prism diopters in patients who underwent IM-OD and ML-OD, respectively. In the IM-OD group, abduction and elevation worsened at the first (*p*<0.05) and third (*p*<0.05) postoperative visits but were restored at 6 months. The versions did not change postoperatively with ML-OD. The preoperative CT-measured medial rectus muscle area predicted new-onset strabismus (*p*=0.023). Significant postoperative medial rectus muscle enlargement occurred in both groups (*p*<0.001). Restriction in elevation and abduction was significantly associated with enlarged inferior (*p*=0.007) and medial rectus muscle areas (*p*=0.002).

**CONCLUSIONS:**

IM-OD is as safe as ML-OD with regard to new-onset strabismus, and represents a good alternative for patients who do not require significant exophthalmos reduction. ML-OD offers greater exophthalmos reduction and smoother postoperative recovery. Patients with preoperative enlarged medial rectus muscle on CT are at risk for new-onset esotropia, and preoperative esotropia is likely to increase after OD.

## INTRODUCTION

Graves’ orbitopathy (GO) covers an array of orbital and periocular changes associated with dysthyroid autoimmune disease, and can lead to muscle and fat expansion, orbital tissue fibrosis, and restriction of extraocular motility, which occur in a self-limited and highly heterogeneous manner (1). Following an active phase, GO becomes inactive (burn-out phase), but remission is rarely complete (2). The disease significantly affects patients’ quality of life due to disfigurement and functional limitations, including exophthalmos, diplopia, and dysthyroid optic neuropathy (3).

Orbital decompression (OD), a critical procedure in the multi-stage rehabilitation of patients with sequelae from GO, can restore premorbid function and appearance (4). Several surgical techniques are currently in use, including lateral wall OD, inferior and medial wall OD, balanced (medial and deep lateral walls) OD, and three-wall OD. This procedure increases orbital capacity with or without orbital fat removal (5). Since its introduction, technical advancements have made the procedure more time-efficient, and incisions are now smaller, postoperative recovery is faster, exophthalmos reduction can be tailored, and most importantly, the incidence of postoperative strabismus has decreased dramatically (6). Preoperative extraocular muscle enlargement is considered a strong predictor of postoperative strabismus (4,7). However, due to the scarcity of prospective randomized controlled trials, the association between OD techniques and the development of postoperative strabismus remains unclear (8).

Based on a comprehensive qualitative and quantitative assessment of extraocular motility and recti muscle measurements on computed tomography (CT) of the orbits, we conducted a prospective randomized clinical trial to compare exophthalmos reduction and determine the effect on ocular motility of two well-established OD techniques: inferomedial wall OD (IM-OD) and balanced medial plus lateral wall orbital decompression (ML-OD).

## METHODS

### Study design

A prospective randomized clinical trial was conducted from 2015 to 2018 at a single referral outpatient ophthalmology service. The study protocol followed the tenets of the Declaration of Helsinki, and Institutional Review Board approval was obtained. All participants provided written informed consent.

### Subjects

Forty-two patients in the inactive phase of GO with clinical indications for OD were studied. GO was classified according to disease activity based on the Clinical Activity Score (CAS) (9). Patients with a CAS <3, who had been clinically stable for at least 6 months, and who had a disease duration of >2 years were considered to be in the inactive phase.

The inclusion criteria were as follows: i) diagnosis of GO in the inactive phase, ii) provision of informed written consent, iii) age ≥21 years, iv) euthyroidism, v) exophthalmometry ≥20 mm, vi) absence of eye abnormalities such as degenerative myopia, microphthalmos, and anophthalmia, vii) absence of other orbital abnormalities such as previous fractures and congenital defects, viii) cooperation with study procedures, ix) ability to comply with the consultation schedule, and x) absence of contraindications for OD in the preoperative clinical evaluation.

The exclusion criteria were as follows: i) myasthenia gravis, ii) pregnancy, iii) previous orbital, strabismus, or eyelid surgery, and iv) other abnormal eye conditions or symptoms preventing the patient from participating in the study, as per the investigator’s clinical judgment.

### Randomization

Patients were randomly assigned to one of two groups according to the surgical technique (IM-OD or ML-OD), by drawing of lots. The researcher scheduling the surgery was different from the orbit surgeons and the researcher performing the preoperative and postoperative evaluations and taking the photographs. The latter researcher and patients were blinded to the OD technique.

### Surgical techniques

IM-OD was performed by one of the authors (R.B.M.), and ML-OD was performed by another author (A.C.P.G.). Both orbit surgeons have extensive experience with their respective techniques. Both techniques were performed under general anesthesia.

IM-OD was performed with some modifications to the previous descriptions (10,11). Briefly, the transcaruncular approach was used to access the medial wall of the orbit (12). A C-shaped incision was made vertically just behind the caruncle in the medial conjunctiva, with dissection posteriorly through the subconjunctival tissue and then medially in the preseptal plane to the posterior lacrimal crest. The medial wall (the lamina papyracea of the ethmoid bone) was completely dissected and fractured, respecting its superior limit with the frontal bone and posterior limit with the lesser wing of the sphenoid bone. The inferior limit (the junction with the maxillary bone comprising the inferomedial orbital strut) was preserved in the anterior portion. The orbital floor (maxillary sinus roof) was accessed through a fornix transconjunctival incision (10,11), without lateral canthotomy whenever possible. The maxillary fracture comprised only the medial portion relative to the infraorbital groove. The periorbita opening was carefully planned to avoid recti muscle paths.

The ML-OD technique consisted of medial and lateral wall decompression while sparing the orbital floor. As in IM-OD, the transcaruncular approach was used to access the medial wall. Access to the lateral wall was achieved as described previously (13,14). The superolateral orbital rim was exposed via lateral incision of the upper eyelid. The lateral wall was dissected, and all three areas of the thick bone were sculpted and thinned using a high-speed diamond drill: the lacrimal fossa (to improve visualization), the greater wing of the sphenoid, and inferolaterally, the zygomatic bone and part of the maxilla. Subsequently, periorbita incisions were made to enable orbital fat herniate into the newly created space.

### Pre- and postoperative evaluation

Before surgery, and 1, 3, and 6 months postoperatively, the patients underwent a complete ophthalmologic examination, including Hertel exophthalmometry and full extrinsic ocular motility (EOM) assessment.

The EOM assessment consisted of measuring the angle of ocular deviation in prism diopters (PD) using the prism and alternate cover test with far and near fixation on Snellen optotypes. In cases of marked ocular motility restriction, deviation measurement was quantified using the Krimsky method. The versions were graded from −1 to −4 to qualify underaction and from +1 to +4 to qualify the overaction of each muscle in its field of action. Normal versions were observed as 0. The presence or absence of binocular diplopia in the nine gaze positions was scored from 0 to 100 using the Diplopia Questionnaire developed by Holmes et al. (15). The presence of torsional diplopia was quantified in degrees using single and double Maddox rod tests in the primary gaze position. Stereopsis was determined using the Titmus stereoacuity test.

### Digital photography

**Figure 1:**
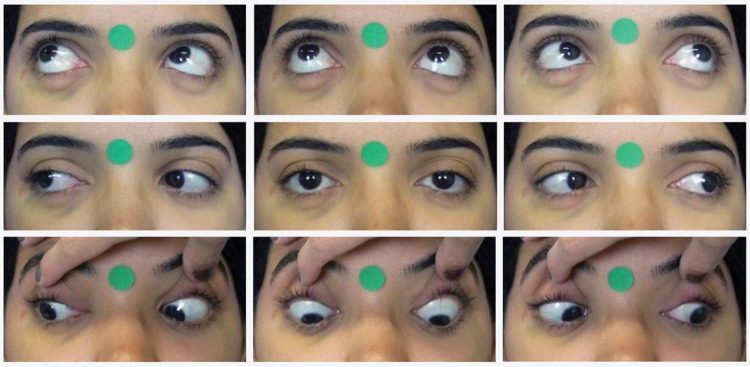
Photographs in the nine positions of gaze.

Standardized frontal photographs (Canon PowerShot SX530 HS) of each participant were taken by a single trained ophthalmologist, both preoperatively and 6 months after surgery. The patient was positioned in a chair 50 cm away from the camera lens. With the head properly aligned, photographs were taken in the nine cardinal positions of gaze. Verbal encouragement was given to ensure head stability and maximum effort toward the extremes of gaze. The photographs were repeated in case of inappropriate movements. In the infraversions, the eyelids were pulled for better observation. The photographs included a 12-mm circular sticker for digital calibration (Figure 1).

### Digital photographic measurements

**Figure 2:**
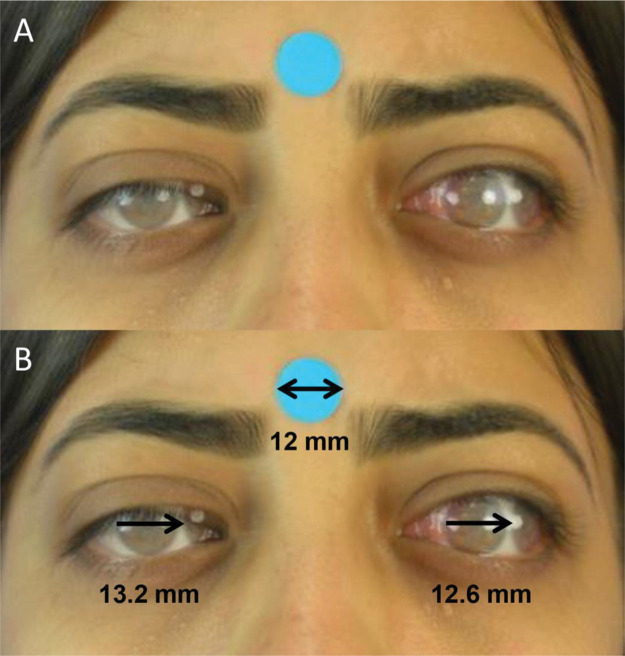
**A.** Juxtaposition of semitransparent images of primary gaze and levoversion (Photoshop) for quantitative version evaluation.**B.** Evaluation of levoversion. Right eye: In adduction, the distance between the lateral limbi of the juxtaposed photos is measured (ImageJ). Left eye: In abduction, the distance between the medial limbi is measured (ImageJ).

Digital images were processed and analyzed by a single researcher using the method proposed by Lim et al. (16). Using Photoshop software (Adobe, San Jose, CA, USA, version 19.1.9), semitransparent photographs of the patient’s versions were successively juxtaposed on a photograph in the primary gaze position (Figure 2A). Later, the distance (mm) between the limbi of the overlapping photographs was measured with the support of ImageJ software (National Institutes of Health, Bethesda, MD, USA, v. 1.52a) (Figure 2B). The calibration of digital measurements was carried out using a 12-mm circular sticker as a reference.

### Computed tomography of the orbits

All participants underwent multi-detector CT of the orbits (Brilliance 16; Philips Medical Systems, Holland) without intravenous contrast within 2 weeks of the preoperative consultation and at the 6-month postoperative visit.

The CT images were obtained through continuous axial sections with the patient lying supine and with the head positioned parallel to the Frankfurt plane. The acquisition parameters were as follows: 120 kV, 200 mA, detector with 16×0.75 mm configuration, 1.5-mm cutting thickness, and 0.7 mm increment.

**Figure 3:**
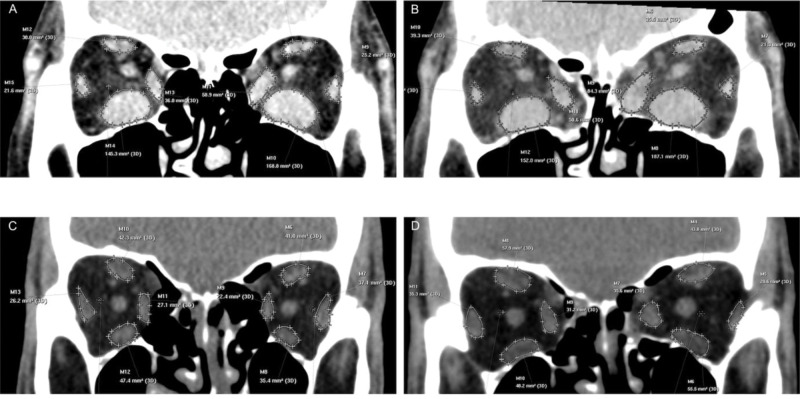
Computed tomography measurements of the recti muscle areas. **A** and **B.** Preoperative (A) and postoperative (B) measurements of a patient submitted to inferomedial wall orbital decompression (IM-OD). **C** and **D.** Preoperative (C) and postoperative (D) measurements of a patient submitted to balanced medial plus lateral wall orbital decompression (ML-OD).

The images were processed at a dedicated CT scanner workstation and evaluated by a single radiologist who was blinded to the surgical technique. In the pre and postoperative images, the area of the recti muscles was measured 9 mm posteriorly to the lateral orbital rim, in a coronal section, with each rectus muscle outlined on the computer screen (Figure 3) (4).

### Statistical analysis

Statistical analysis was performed using the Stata software (StataCorp, College Station, TX, USA, version 15) and Statistica (TIBCO Software Inc., Palo Alto, CA, USA, version 13).

The sample size was established based on exophthalmos reduction as the main variable. The mean standard deviation found in the literature is 2.1 mm, and the desired effect size, based on clinical judgment, is 1.5 mm; the minimum sample size of 24 eyes in each group was reached.

The chi-square association test was used to verify the equivalent distributions of the demographic and clinical variables. Repeated-measures analysis of variance was used to calculate differences between the two groups with regard to the mean preoperative and postoperative quantitative parameters. Multiple comparisons were performed using the Tukey HSD test, when appropriate. Alternatively, when the variances were not homogeneous (compared with the Levene test), the Friedman test was used. The McNemar’s test was used to verify the frequency of categorical variables. In all analyses, differences were considered statistically significant when *p*<0.05 (alpha error=5%), with *p*<0.001 regarded as highly significant.

## RESULTS

### Demographic and clinical variables

Table 1 shows the demographic data and clinical characteristics of the 42 patients included in the study. No statistically significant differences were observed between the groups regarding sex distribution, age, TRAb dosage, smoking, family history of thyroid disease, treatment for Graves’ disease, or treatment for GO.

No major surgical complications (*e.g.*, visual loss, permanent infraorbital dysesthesia, and hypoglobus) occurred in either group.

### Exophthalmos

Twenty-one patients (42 orbits) underwent IM-OD, and 21 (42 orbits) underwent ML-OD. The mean preoperative exophthalmometry findings were similar between the two groups (*p*=0.899). Postoperative reduction on exophthalmometry was significant in both groups (*p*<0.001), but significantly greater with ML-OD than with IM-OD (3.8±3.1 mm *vs.* 2.4±1.9 mm; *p*=0.010) (Table 2).

### Strabismus and diplopia

In the IM-OD group, 88.9% of the patients remained orthotropic during the postoperative period. All patients with preoperative strabismus (14.2%) were esotropic and experienced a postoperative increase in the horizontal deviation angle of 24±6 PD. Two of these patients had associated vertical strabismus, which remained unaffected by the OD.

In the ML-OD group, 76.5% of patients remained without strabismus during the postoperative period. In one of the four patients who had strabismus before surgery (19%), deviation improved (the patient had a history of exotropia), while, in the other three patients (who had a history of esotropia), the horizontal deviation angle increased by 12±8.8 PD after OD. One of these patients had associated vertical strabismus, which remained unaffected by the OD.

New-onset strabismus was observed in two patients (11.1%) who underwent IM-OD, and four patients (23.5%) underwent ML-OD. All six patients developed esotropia (Table 3). The McNemar’s test revealed no statistically significant difference in the frequency of pre and postoperative strabismus (IM-OD *p*=0.500; ML-OD *p*=0.219) or stereopsis (IM-OD *p*=0.317; ML-OD *p*=0.564) in either group.

The Diplopia Questionnaire scores were statistically similar pre and postoperatively, and between the two groups (*p*=0.094). Similarly, no statistically significant change was found for torsional diplopia (*p*=0.386 for the Maddox rod test; *p*=1.000 for the double Maddox rod test) before and after surgery and between the two OD techniques.

### Versions evaluation

In the qualitative clinical evaluation of the versions, temporary postoperative worsening of versions was observed in the IM-OD group. Thus, abduction, elevation in abduction, elevation, and elevation in adduction worsened in the first (*p*<0.05) and third (*p*<0.05) postoperative months. However, at 6 months, the patient returned to the preoperative status. Versions did not change postoperatively in the ML-OD group.

This behavior was also reflected in the quantitative photographic analysis: versions worsened temporarily in terms of abduction and elevation in abduction in the IM-OD group, but not in the ML-OD group (Table 4).

### CT muscle evaluation

Preoperatively, the recti muscle areas were similar for both the IM-OD and ML-OD groups (inferior rectus, *p*=0.076; medial rectus, *p*=0.230; superior rectus, *p*=0.063; lateral rectus, *p*=0.200). The preoperative medial rectus muscle area was predictive of new-onset strabismus in our sample (*p*=0.023). The inferior rectus muscle area (*p*=0.007) and medial rectus muscle area (*p*<0.001) were larger in patients with preoperative strabismus than in orthotropic patients.

Postoperatively, the medial rectus muscle area was significantly enlarged in both groups (*p*<0.001). No statistically significant enlargement of the inferior rectus muscle was observed after OD in either group (IM-OD, *p*=0,163; ML-OD, *p*=0,681). Patients with larger inferior rectus muscle area on CT had restriction in elevation (*p*=0.007), while a large medial rectus muscle area was associated with restriction in abduction (*p*=0.002).

## DISCUSSION

OD is performed by removing one or more orbital walls, and in some techniques, orbital fat. Most commonly, this involves the lateral wall, medial wall, and orbital floor, in several combinations, and with different approaches. Technical and conceptual advances have improved the surgical outcomes of this procedure (6,17). Considering the low morbidity of modern OD techniques, indications have been expanded to include esthetic-functional deformities. In this context, the pursuit of safe, cost-effective, customized, and minimally invasive techniques is paramount (2).

The widely employed IM-OD technique allows access to the area of interest through hidden conjunctival incisions. Moreover, the technique is fast, low-cost, and in many cases, provides satisfactory exophthalmos reduction. The relatively high postoperative diplopia rates reported for this technique are usually associated with transantral approaches. Indeed, McCord (18) found a significantly lower incidence of strabismus with the transpalpebral approach (6%) than with the transantral approach (41%). Concerns about ocular globe dystopia and strabismus have led many surgeons to spare the anterior inferomedial orbital strut; however, the gain in safety comes at the cost of effectiveness in exophthalmos reduction.

Balanced OD (ML-OD) has gained popularity due to the reportedly lower risk of diplopia and hypoglobus (19). Several authors have argued that the removal of the orbital wall close to a restricted rectus muscle may lead to ocular motility imbalance, and consequently, postoperative new-onset or worsening of diplopia. As the inferior rectus muscle is most frequently involved in GO, the orbital floor should be avoided (20). The medial wall may be accessed transnasally or more swiftly through a transcaruncular incision, with direct visualization. The removal of the lateral wall in the ML-OD allows for a significant reduction in axial exophthalmos. Lateral wall approach involves removing portions of the frontal bone, the zygomatic bone, and the greater wing of the sphenoid, while deep lateral wall OD includes the removal of the diploe of the greater wing of the sphenoid. Minimally invasive *ab interno* lateral wall OD without lateral canthotomy or osteotomy is associated with fewer complications and quicker recovery (14).

In our study, both techniques effectively reduced exophthalmos, but ML-OD (3.8±3.1 mm) was significantly more efficient than IM-OD (2.4±1.9 mm). Despite the minimally invasive lateral wall approach in ML-OD, exophthalmos reduction was similar to that achieved in earlier studies (21). On the other hand, the results obtained with IM-OD compared unfavorably to those of other reports (5), possibly due to the preservation of the anterior portion of the inferomedial orbital strut. It should be stressed that to prevent ocular motility imbalance, no fat debulk was performed in our patients, regardless of the technique employed.

The development of postoperative strabismus following OD is a multifactorial phenomenon. In addition to preoperative extraocular muscle size (4,7), other factors that require consideration include surgical complications, the extent of bone removal and periorbital opening, asymmetrical orbital wall removal, inferomedial orbital strut status, fat removal, and surgical skill and experience. These multiple factors make it difficult to determine the actual effect of different surgical techniques on the onset of strabismus. A large body of research on OD in patients with GO is available, but few studies have performed standardized evaluations of surgical outcomes (5,22). Indeed, most are retrospective studies focused on a single technique or different indications for surgery, and many have poorly matched groups (23). Although several multicenter studies on different techniques have been conducted, the results are inconsistent (5). The assessment of diplopia is also critical, with some authors stressing the importance of formal orthoptic evaluations (24).

Our prospective randomized clinical trial compared similar groups of patients with no statistically significant differences in clinical status, disease activity, surgical indication, or CT-measured recti muscle areas. The surgeries were performed in a consistent manner by experienced surgeons, with no complications such as hemorrhage, rectus muscle injury, or cerebrospinal fluid leak. The inferomedial orbital strut was partially preserved in the IM-OD group (anterior portion), and was completely preserved in the ML-OD group. A judicious opening of the periorbita was performed in both groups, and no orbital fat was debulked. Having controlled for all these variables, IM-OD and ML-OD were found to be equally safe, with low rates of new-onset strabismus. A similar trend was observed in another cohort study (23).

Mainville and Jordan (24) reported a preoperative strabismus prevalence of 26% and a postoperative prevalence of 40.7%. In our study, the preoperative prevalence was 14.2% (IM-OD) and 19% (ML-OD), and the postoperative prevalence was 23.8% and 33.3%, respectively. Postoperative new-onset strabismus occurred in two patients (11.1%) in the IM-OD group and four patients (23.5%) in the ML-OD group. All six patients progressed to esotropia, which was in agreement with the available literature (7,19,24,25). Graham et al. (26) found a particularly low rate (10%) of new-onset diplopia in a cohort of 40 patients who underwent ML-OD.

The resolution of strabismus after OD has been reported in several studies based on small samples, and with little or no information provided on strabismus type and evaluation methods (19,24,27). In our investigation, a single patient (1/42) with preoperative exotropia submitted to ML-OD experienced resolution of diplopia (esotropic shift). Based on this finding and the available literature, improvement in diplopia is uncommon after OD (5,28).

With a mean increase of 24±6.9 PD (IM-OD) and 12±8.8 PD (ML-OD), deviation worsened in patients with preoperative esotropia. Fabian et al. (25) observed a mean increase in esotropia of approximately 12 PD. Consistent with other reports, the esotropic shift appears to be the most frequent ocular motility disturbance after OD (7,25,28,29).

The preoperative and postoperative scores of the Diplopia Questionnaire (15) were statistically similar regardless of the OD technique employed. As the quantification of diplopia relies on gaze position and not on deviation size, diplopia scores were unaffected by the observed postoperative increase in deviation. In addition, although reported by other researchers (30), none of our patients had torsional strabismus.

Ocular version assessment is an essential part of EOM assessments, and several methods have been proposed (31). To ensure accuracy, we performed routine qualitative clinical evaluations and a quantitative photographic method (16). In previous studies, neither technique was associated with late postoperative changes in versions (28,32). Patients who underwent IM-OD (but not ML-OD) experienced transient worsening of versions (abduction and elevation) in the early postoperative months, with similar findings reported in other studies (29). Thus, setting aside the question of clinical relevance, ML-OD appears to provide a smoother recovery than IM-OD.

Similar to the findings of Eing et al. (4) and Nunery et al. (7), medial rectus muscle size was predictive of new-onset strabismus (esotropia). Furthermore, the inferior and medial rectus muscle areas were larger in patients with preoperative strabismus than in orthotropic patients. The significant postoperative increase in the recti muscle areas observed with both OD techniques was associated with greater restrictions in elevation (inferior rectus) and abduction (medial rectus). An increased medial rectus muscle area is an expected finding after medial wall OD (33), although the exact reason for this is unclear. OD-related reactivation of GO is rare, and, as expected, none of our patients had clinical evidence of postoperative reactivation. Following OD, the medial rectus muscle moved into the ethmoidal sinus cavity. This change in position, associated with reduced pressure in the orbit, could lead to changes in the intermuscular connective tissue and in other connections between the extraocular muscles and orbital contents, which could allow the extraocular muscles to increase in size. Venous stasis, a known cause of extraocular muscle enlargement, may also be involved (33,34). This enlargement of the medial rectus muscle area may explain why the postoperative strabismus was esotropia in all cases. Zloto et al. (35) also found medial wall OD to be associated with an esotropic shift, and hypothesized that medial orbital expansion enables the medial rectus muscle to expand more medially, causing esotropia by worsening the restriction to abduction.

## CONCLUSIONS

IM-OD is efficient in reducing moderate exophthalmos and is safe for postoperative maintenance of ocular alignment and stereoacuity. However, ML-OD is more efficient in exophthalmos reduction, with low rates of postoperative strabismus and smoother recovery with regard to eye movement restrictions.

Esotropia is the most common type of new-onset strabismus. Preoperative esotropia increased after OD, but the vertical strabismus remained unchanged. Therefore, patients with preoperative esotropia should be informed about the risk of worsening deviation and the possible need for surgical correction of strabismus. They should also be made aware that the resolution of diplopia is not an expected outcome of OD.

Preoperative medial rectus muscle size was predictive of new-onset strabismus. The inferior and medial rectus muscle areas were larger in patients with preoperative strabismus than in orthotropic patients. Postoperative enlargement of the medial rectus muscle correlated with eye movement restrictions. Our findings may help orbit surgeons select an appropriate OD technique and identify patients with GO at risk for new-onset or worsening of strabismus.

## AUTHOR CONTRIBUTIONS

Leite CA, Pereira TS, Chiang J and Moritz RB participated in the acquisition, analysis, and interpretation of the data. Leite CA, Gonçalves ACP and Monteiro MLR provided substantial contributions to the conception and design of the study, interpretation of results, and manuscript drafting.

## Figures and Tables

**Table 1:** Demographic and clinical variables of patients with GO submitted to OD.

		Group (%)		
Variable	Category	IM-OD (n=21)	ML-OD (n=21)	*p*-value*
Sex	Female	15 (71.4)	16 (76.2)	0.726
	Male	6 (28.6)	5 (23.8)	
Age (years±SD)		47.5±12.7	49.9±10.9	0.616
TRAb	Positive	7 (43.8)	8 (47.1)	0.849
Smoking	Yes	9 (42.9)	12 (57.1)	0.355
Family history	Yes	11 (52.4)	8 (38.1)	0.352
**Treatment for GD**				
Anti-thyroid drugs	Yes	17 (81.0)	18 (85.7)	1.000
Radioiodine therapy	Yes	11 (52.4)	14 (66.7)	0.346
Thyroidectomy	Yes	2 (9.5)	7 (33.3)	0.130
Hormonal reposition	Yes	2 (9.5)	0 (0.0)	0.488
**Treatment for GO**				
Lubricant eye drops	Yes	14 (66.7)	17 (80.9)	0.292
Corticosteroids	Yes	20 (95.2)	20 (95.2)	1.000
Radiotherapy	Yes	1 (4.8)	1 (4.8)	1.000

* Chi-square association test. TRAb: Anti-TSH receptor antibody, GD: Graves’ disease, GO: Graves’ orbitopathy, IM-OD: Inferomedial wall orbital decompression, ML-OD: Balanced medial plus lateral wall orbital decompression.

**Table 2:** Hertel exophthalmometry findings in the two study groups (ML-OD and IM-OD) before and after orbital decompression.

		Exophthalmometry (mm) Mean±SD (range)			
Group	n	Preoperative	Postoperative	Exophthalmos reduction (mm) Mean±SD (range)	*p-*value
IM-OD	42	23.9±2.8 (20-30)	21.4±2.9 (14-28)	2.4±1.9 (0.5-8)	<0.001*
ML-OD	42	23.5±2.6 (20-34)	19.6±2.2 (14-24)	3.8±3.1 (0.5-12)	<0.001*
		*p=*0.899*	*p=*0.010*		

* Repeated-measures analysis of variance/Tukey-HSD test, IM-OD: Inferomedial wall orbital decompression, ML-OD: Balanced medial plus lateral wall orbital decompression.

**Table 3:** Prevalence and incidence of strabismus and diplopia in patients with Graves’ orbitopathy submitted to OD.

Group	Strabismus/diplopia	n	%
IM-OD (n=21)	Preoperative	3	14.2 (3/21)
	Postoperative	5	23.8 (5/21)
	New-onset strabismus	2	11.1 (2/18)
	Strabismus improvement	0	0
ML-OD (n=21)	Preoperative	4	19 (4/21)
	Postoperative	7	33.3 (7/21)
	New-onset strabismus	4	23.5 (4/17)
	Strabismus improvement	1	25 (1/4)

IM-OD: Inferomedial wall orbital decompression, ML-OD: Balanced medial plus lateral wall orbital decompression.

**Table 4:** Quantitative measurements of ocular versions in patients with Graves’ orbitopathy before and 6 months after OD.

		Quantitative evaluation (mm) Mean (SD)		
Version	Group	Preoperative	Postoperative	*p*-value^Ŧ^
Abduction**	IM-OD	8.5 (1.8)	7.4 (2.2)	**0.023**
	ML-OD	7.9 (2.4)	7.1 (2.3)	0.126
Adduction*	IM-OD	7.3 (2.0)	7.5 (2.5)	0.540
	ML-OD	7.2 (2.5)	6.6 (2.3)	0.294
Elevation in abduction**	IM-OD	6.7 (3.3)	5.4 (3.3)	**0.024**
	ML-OD	5.9 (3.5)	4.9 (2.7)	0.885
Elevation*	IM-OD	4.7 (2.5)	4.1 (2.3)	0.210
	ML-OD	4.0 (2.1)	3.5 (2.2)	0.439
Elevation in adduction*	IM-OD	5.9 (2.6)	4.8 (3.0)	0.111
	ML-OD	5.9 (3.0)	4.9 (2.4)	0.220
Depression in abduction**	IM-OD	10.2 (2.0)	9.7 (2.6)	0.624
	ML-OD	9.5 (2.2)	9.8 (1.4)	0.903
Depression*	IM-OD	9.8 (1.9)	9.3 (2.1)	0.805
	ML-OD	9.8 (1.9)	9.3 (1.9)	0.972
Depression in adduction**	IM-OD	10.6 (2.3)	10.4 (2.7)	0.201
	ML-OD	9.8 (2.5)	10.7 (1.6)	0.210

* Repeated-measures analysis of variance

** Friedman test, **^Ŧ^** *post-hoc* test (Tukey-HSD), IM-OD: Inferomedial wall orbital decompression, ML-OD: Balanced medial plus lateral wall orbital decompression.
